# Multi-Country Evaluation of the Sensitivity and Specificity of Two Commercially-Available NS1 ELISA Assays for Dengue Diagnosis

**DOI:** 10.1371/journal.pntd.0000811

**Published:** 2010-08-31

**Authors:** Maria G. Guzman, Thomas Jaenisch, Roger Gaczkowski, Vo Thi Ty Hang, Shamala Devi Sekaran, Axel Kroeger, Susana Vazquez, Didye Ruiz, Eric Martinez, Juan C. Mercado, Angel Balmaseda, Eva Harris, Efren Dimano, Prisca Susan A. Leano, Sutee Yoksan, Elci Villegas, Herminia Benduzu, Iris Villalobos, Jeremy Farrar, Cameron P. Simmons

**Affiliations:** 1 Virology Department, Pan American Health Organization/World Health Organization Collaborating Center for the Study of Dengue and Its Vector, Instituto de Medicina Tropical “Pedro Kouri”, Habana, Cuba; 2 Section Clinical Tropical Medicine, Department of Infectious Diseases, University Hospital of Heidelberg, Heidelberg, Germany; 3 Oxford University, Clinical Research Unit, Ho Chi Minh City, Vietnam; 4 Department of Medical Microbiology, Faculty of Medicine, University of Malaya, Kuala Lumpur, Malaysia; 5 TDR-World Health Organization, Geneva, Switzerland; 6 CNDR, Managua, Nicaragua; 7 Division of Infectious Diseases and Vaccinology, School of Public Health, University of California, Berkeley, California, United States of America; 8 San Lazaro Hospital, Manila, Philippines; 9 Mahidol University, Bangkok, Thailand; 10 Instituto Experimental “Jose W Torrealba” Núcleo Universitario “Rafael Rangel”, Universidad de los Andes Trujillo, Bogotá, Venezuela; 11 Hospital Central de Maracay, Maracay, Venezuela; Centers for Disease Control and Prevention, United States of America

## Abstract

**Background:**

Early diagnosis of dengue can assist patient triage and management and prevent unnecessary treatments and interventions. Commercially available assays that detect the dengue virus protein NS1 in the plasma/serum of patients offers the possibility of early and rapid diagnosis.

**Methodology/Principal Findings:**

The sensitivity and specificity of the Pan-E Dengue Early ELISA and the Platelia™ Dengue NS1 Ag assays were compared against a reference diagnosis in 1385 patients in 6 countries in Asia and the Americas. Platelia was more sensitive (66%) than Pan-E (52%) in confirmed dengue cases. Sensitivity varied by geographic region, with both assays generally being more sensitive in patients from SE Asia than the Americas. Both kits were more sensitive for specimens collected within the first few days of illness onset relative to later time points. Pan-E and Platelia were both 100% specific in febrile patients without evidence of acute dengue. In patients with other confirmed diagnoses and healthy blood donors, Platelia was more specific (100%) than Pan-E (90%). For Platelia, when either the NS1 test or the IgM test on the acute sample was positive, the sensitivity versus the reference result was 82% in samples collected in the first four days of fever. NS1 sensitivity was not associated to disease severity (DF or DHF) in the Platelia test, whereas a trend for higher sensitivity in DHF cases was seen in the Pan-E test (however combined with lower overall sensitivity).

**Conclusions/Significance:**

Collectively, this multi-country study suggests that the best performing NS1 assay (Platelia) had moderate sensitivity (median 64%, range 34–76%) and high specificity (100%) for the diagnosis of dengue. The poor sensitivity of the evaluated assays in some geographical regions suggests further assessments are needed. The combination of NS1 and IgM detection in samples collected in the first few days of fever increased the overall dengue diagnostic sensitivity.

## Author Summary

Dengue is the most important mosquito-borne viral disease of humans and an enormous public health burden in affected countries. Early, sensitive and specific diagnosis of dengue is needed for appropriate patient management as well as for early epidemic detection. Commercially available assays that detect the dengue virus protein NS1 in the plasma/serum of patients offer the possibility of early and rapid diagnosis. Here we evaluated two commercially available ELISA kits for NS1 detection (Pan-E Dengue Early ELISA and the Platelia™ Dengue NS1 Ag). Results were compared against a reference diagnosis in 1385 patients in 6 countries in Asia and the Americas. Collectively, this multi-country study suggests that the best performing NS1 assay (Platelia) had moderate sensitivity (median 64%, range 34–76%) and high specificity (100%) for the diagnosis of dengue. The combination of NS1 and IgM detection in samples collected in the first few days of fever increased the overall dengue diagnostic sensitivity.

## Introduction

Dengue is the most important mosquito-borne viral disease of humans and an enormous public health burden in affected countries. An estimated 50–100 million dengue cases occur annually, including 250,000–500,000 cases of severe illness and around 25,000 deaths. Approximately 2.5 billion people live in dengue endemic countries and the illness is reported in Southeast Asia, Western Pacific, the Americas, Africa and Mediterranean regions [1]–[3].

Dengue viruses (DENVs), of which there are four serotypes, cause a variable spectrum of disease that ranges from an undifferentiated fever to dengue fever (DF) through to more severe syndromes called dengue haemorrhagic fever (DHF) and dengue shock syndrome (DSS). DHF/DSS is a vasculopathy characterized by capillary leakage and haematological dysregulation. There are no licensed vaccines or specific antiviral therapies for dengue, and patient management relies on good supportive care.

Early, sensitive and specific diagnosis of dengue can assist in patient triage and for those who require it, early supportive management. In principle, early diagnosis could also facilitate timely public health interventions, e.g. vector control targeted at the households of index cases. Existing approaches to dengue diagnosis rely primarily on detection of DENV-reactive IgM; in more specialised settings this is augmented with detection of DENV RNA using home made RT-PCR or rarely, virus isolation [4], [5]. Whilst generally robust, a limitation of IgM-based diagnostic approaches is poor sensitivity in the first few days of illness and in some settings, serological cross-reactivity with other Flaviviruses [4], [5]. Recently, the diagnostic accuracy of commercial diagnostic assays that detect the DENV NS1 protein in plasma/serum samples have been described [6]–[13]. NS1 is a 55kDa glycoprotein secreted by DENV infected cells “in vitro” and “in vivo”. Whilst the role of NS1 in DENV biology is not well understood, high plasma NS1 concentrations early in illness have been associated with more severe disease [14], [15]. The targeting of NS1 in diagnostic assays potentially offers the opportunity for an early, specific diagnosis of DENV infection since it can be detected prior to the appearance of measurable DENV-reactive IgM [8]. Whilst NS1 is a promising diagnostic target, the assessment of currently available NS1 assays across a breadth of patient populations, viral serotypes and lineages is important in evaluating where and when these assays [16] may fit into the laboratory diagnosis of dengue.

At the end of 2006, the Dengue Scientific Working Group under the leadership of the World Health Organization Special Programme for Research and Training in Tropical Diseases (WHO/TDR) established priorities for dengue research aimed at improving dengue treatment, prevention and control. The evaluation of new diagnostics were included among these priorities [17], [18]. To this end, the purpose of the current study was to assess the sensitivity and specificity of two commercial NS1 assays in six countries.

## Materials and Methods

### The DENCO study

The DENCO project was a multi-centre prospective observational study of dengue in Southeast Asia (Malaysia, Thailand, The Philippines and Vietnam) and the Americas (Nicaragua and Venezuela). The study sites at which patients were enrolled were: Department of Paediatrics, Faculty of Medicine, University of Malaya, Kuala Lumpur, Malaysia; Queen Sirikit National Institute of Child Health, Bangkok, Thailand; San Lazaro Hospital, Manila, The Philippines; Hospital for Tropical Disease, Ho Chi Minh City, Viet Nam, Children's Hospital #1, Ho Chi Minh City, Viet Nam; Children's Hospital #2, Ho Chi Minh City, Viet Nam; Children's Hospital Manuel Jesus de Rivera, Managua, Nicaragua; Research Centre Jose W. Torrealba, University des Andes, Trujillo and Hospital Central, Maracay, Venezuela.

### Patient enrolment

Following written informed consent by the study participant, or a parent/guardian in the case of children, patients above 6 months of age with clinically suspected dengue and fever for less than 7 days were enrolled in the study. At 5 centres out-patients were recruited as well as in-patients. Patients were followed daily by trained study physicians using standardised case report forms (CRFs) describing clinical, laboratory, diagnostic and management information in detail. Ethical approval was obtained from the Ethics Review Committee of WHO and each institution involved. All patients in these studies were assessed daily by a study physician and had serial haematocrit and platelet estimations performed, as well as appropriate sampling for diagnostic serology and virology. Two plasma or sera samples were collected from each patient, one at day of the enrolment and the second 7–14 days after fever onset. Dengue diagnosis was confirmed by either of the following methods: virus isolation in *Aedes albopictus* cell line (C6/36), by RT/PCR detection as previously described and IgM (MAC-ELISA), IgG (GAC-ELISA or Inhibition ELISA Method, EIM) and total antibody seroconversion (by Hemagglutination Inhibition assay) following the standard procedures at each study site [19]–[30]. The Hemagglutination Inhibition assay was standardized following WHO criteria and WHO recommended cut-off values were utilized [29]. As previously described, RT/PCR methods used here have sensitivity figures from 90 to 100% [20]–[23]. Other investigations and clinical management were at the discretion of the attending physicians. After discharge each patient was classified using the former WHO criteria for DF, DHF and DSS [30]. From November 2007 to January 2008, we prospectively tested acute plasma (or serum) samples from children and adults enrolled in these studies.

### Characteristics of the study population

**Figure 1:**
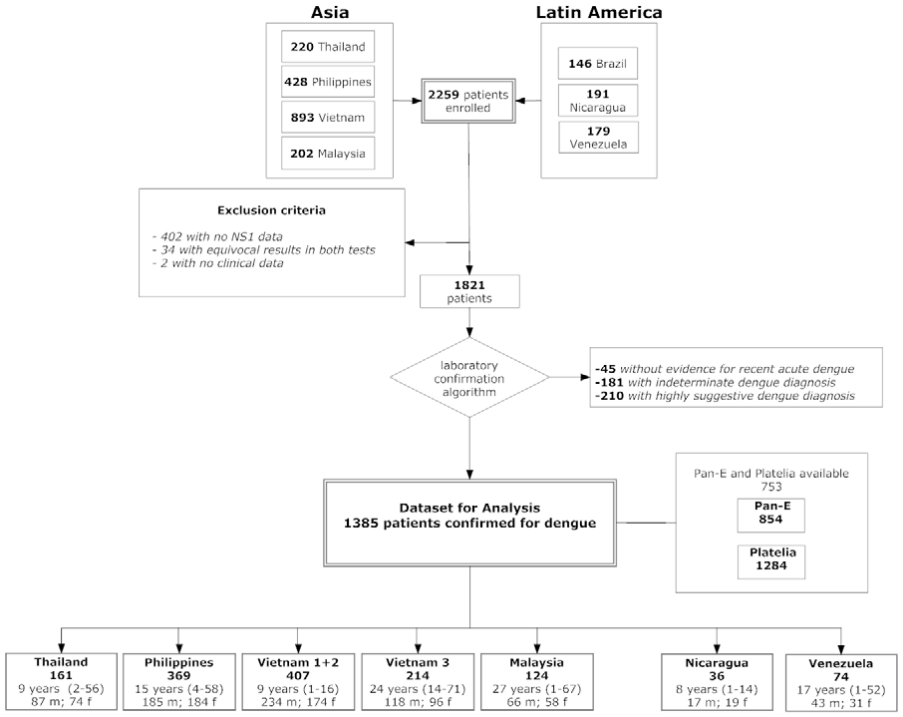
Flow chart summarising multi-country enrolment of dengue patients and basis for the final dataset.

Between August 2006 and May 2007 a total of 2259 patients were recruited to the DENCO study at the 11 participating hospitals. NS1 detection was attempted using at least one of the two NS1 tests in 1821 patients. From amongst the 1821 patients, there were 1385 with laboratory-confirmed dengue and 45 with no laboratory evidence of acute or recent dengue. A further 391 had either indeterminate laboratory results or suggestive serology; results from these cases were not included in the analysis. The flow-chart in Figure 1 summarises the numbers and geography of enrolment and the classification of patients according to the results of reference diagnostic tests including demographic information.

### Laboratory investigations

Serological and virological dengue diagnostics were performed in each participating country according to local protocols, with support provided by WHO designated laboratories as necessary (for participating laboratories see Table 1) [19]–[30]. The definitions employed at each site for “confirmed dengue case” are described in Table 2. For NS1 sensitivity analysis, patients with laboratory confirmation of dengue by serological or virological means were the reference population. For an assessment of NS1 specificity, patients in whom there was no evidence of acute or recent dengue (defined as serologically and virologically negative and in whom there were a minimum of 2 plasma or serum samples tested with the second collected ≥7 days after fever onset and >2 days after the first sample) were studied. As an additional assessment of specificity, two sera panels (one prepared at the Institute of Tropical Medicine “Pedro Kouri” in Cuba and the other at the Mahidol University, Bangkok, Thailand) from healthy individuals and from non-dengue patients were employed.

**Table 1:** List of laboratories performing diagnostic testing for the patients enrolled in the DENCO study.

Location / Country	Hospital	Laboratory
**Bangkok / Thailand**	Queen Sirikit National Institute of Child Health	AFRIMS, Bangkok* – all tests
**Manila / Philippines**	San Lazaro Hospital	AFRIMS certified laboratory at San Lazaro Hospital* for serologyPCR performed at Department of Virology, Nagasaki University, Nagasaki, Japan; serotyping done at San Lazaro Hospital, Manila, biomolecular laboratory (SACCL)
**Ho Chi Minh City / Vietnam**	Children's Hospital No. 1	Oxford University Clinical Research Unit, HCMC** – all tests
	Children's Hospital No. 2	
	Hospital for Tropical Diseases	
**Kuala Lumpur / Malaysia**	University of Malaya Medical Centre, University of Malaya	Department of Medical Microbiology, Faculty of Medicine, University Malaya** - all tests
**Managua / Nicaragua**	Hospital Infantil Manuel de Jesus Rivera La Mascota	Centro Nacional de Diagnóstico y Referencia, Ministry of Health, Managua – all testsIgM, IgG and NS1 repeated at IPK Cuba**
**Maracay & Trujillo/ Venezuela**	Hospital Central de MaracayHospital de Trujillo	Trujillo Hospital Laboratory – all testsIgM, IgG, viral isolation repeated and NS1 done at IPK Cuba**

AFRIMS - Armed Forces Research Institute of Medical Sciences, IPK - Instituto de Medicina Tropical “Pedro Kouri”.

*AFRIMS laboratory network in Asia.

**Member of the Tropical Disease Research –Pediatric Dengue Vaccine Initiative network of proficiency tested laboratories for dengue diagnostic evaluation.

**Table 2:** Laboratory criteria employed at country level for dengue laboratory classification as confirmed dengue case.*

Country	Confirmed dengue case (one of the following)**	Patients without evidence of recent acute dengue (all countries)
All countries	RT-PCR positive or virus isolation positive	Having paired plasma or serum specimens (collected ≥3 days apart) with the last sample collected ≥7 days after illness onset and RT-PCR negative and virus culture negative (at least one of the two being done on the acute sample), and serologically negative in locally used IgM and IgG assays
Thailand, The Philippines (according to AFRIMS protocol)	IgM> = 40 units (acute or convalescent sample or both) and IgG titer increase to above 100 units (paired samples)	
	Twofold IgG titer increase (paired samples) with a titer > = 100 units in the convalescent sample	
Malaysia, Nicaragua, Venezuela, Vietnam	IgM seroconversion (paired samples)	
	IgG seroconversion (paired samples) or fourfold or greater increase in titer (paired samples)	

*For each test validated local protocols were used at each site. Serology results are based on IgM and IgG Capture ELISA of acute and convalescent specimens except where indicated.

**Four laboratories employed the RT/PCR protocol described by Lanciotti, et al., 1992 [20], one employed the protocols by Kong et al., 2006, J Virol Methods and Yong et al., 2007 Singaporean Med J [22], [23],and the other the protocol by Laue et al., . J Clin Microbiol 1999 [21]. All laboratories employed MAC-ELISA. One laboratory employed Inhibition ELISA Method for IgG study while other four used GAC-ELISA. HI: hemagglutination inhibition assay was done in one laboratory (WHO recommendations were followed) [30].

### NS1 detection kits

Pan-E Dengue Early ELISA from Panbio (Brisbane, Australia), (Kit Pan-E) and Platelia Dengue NS1 AG from Bio-Rad (Marnes-la-Coquette, France), (kit Platelia) were evaluated. Both kits are based on a sandwich format microplate enzyme immunoassay for the detection of DENV NS1 employing a peroxidase-labelled murine monoclonal antibodies as probes. Samples were tested for NS1 detection following the manufacturer's recommendations. Sera were classified as NS1 positive, negative and equivocal according to the manufacturer's instructions. For the purposes of analysis, equivocal samples were excluded from the analysis.

### Data management and analysis

Data were double-entered and checked at two established data-entry facilities in Guatemala (Center for Health Studies, Universidad del Valle de Guatemala) and Thailand (WHO/TDR Clinical Data Management Collaborating Center, Faculty of Allied Health Sciences, Thammasat University, Thailand) and the two datasets were subsequently merged. Data analysis was performed at the Section of Clinical Tropical Medicine at the University of Heidelberg, Germany, using STATA versions 9.2 and 10, (STATA corporation, College Park, Texas).

## Results

### Overall sensitivity of NS1 tests versus reference diagnosis of confirmed dengue

**Figure 2:**
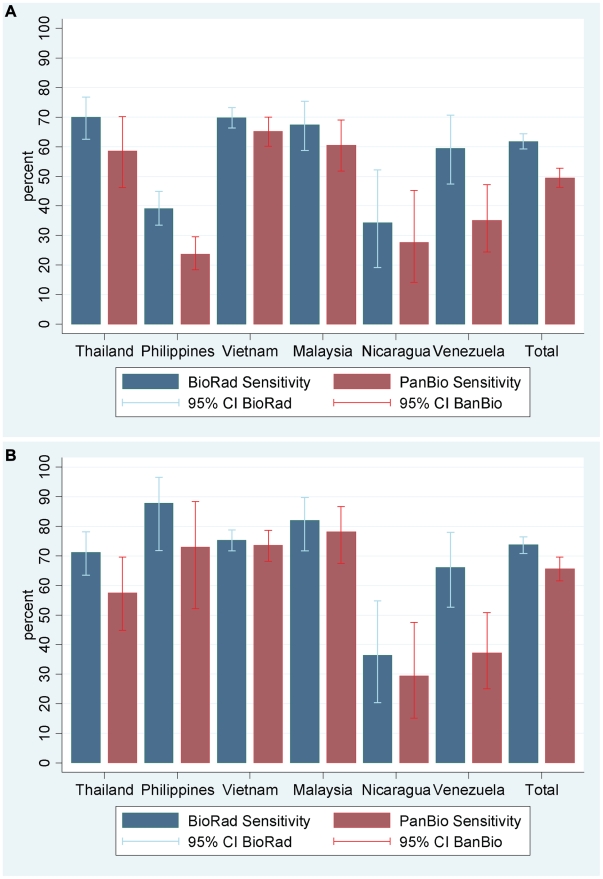
Sensitivity of kits Pan-E and Platelia. Shown are the sensitivities (±95% CI) of kits Pan-E and Platelia assays from six Asian and Latin-American countries in 1385 patients with a laboratory confirmed diagnosis of dengue (A) and sensitivities in the subgroup of 933 patients confirmed by PCR or viral isolation (B).

The diagnostic sensitivity of kits Pan-E and Platelia assays was evaluated in 854 and 1284 serum samples respectively (Figure 1) from patients with a laboratory confirmed dengue diagnosis. Kit Pan-E it could not be performed in all available samples for logistical reasons relating to assay availability at some sites. The sensitivity of the kit Pan-E ranged from 24% in The Philippines to 72% in Vietnam (overall sensitivity rate of 52%). The sensitivity of the kit Platelia ranged from 34% in Nicaragua to 76% in Thailand (overall sensitivity rate of 66%) (Figure 2A).

### NS1 sensitivity in relation to RT/PCR results

Compared to RT/PCR results, sensitivity of kit Pan-E ranged from 29–79% (overall sensitivity rate of 67%; 95% CI 63–71%) and the sensitivity of kit Platelia from 36–88% (overall sensitivity rate of 77%; 95% CI 74–79%) (Figure 2B).

### Sensitivity of NS1 tests by day of illness

**Figure 3:**
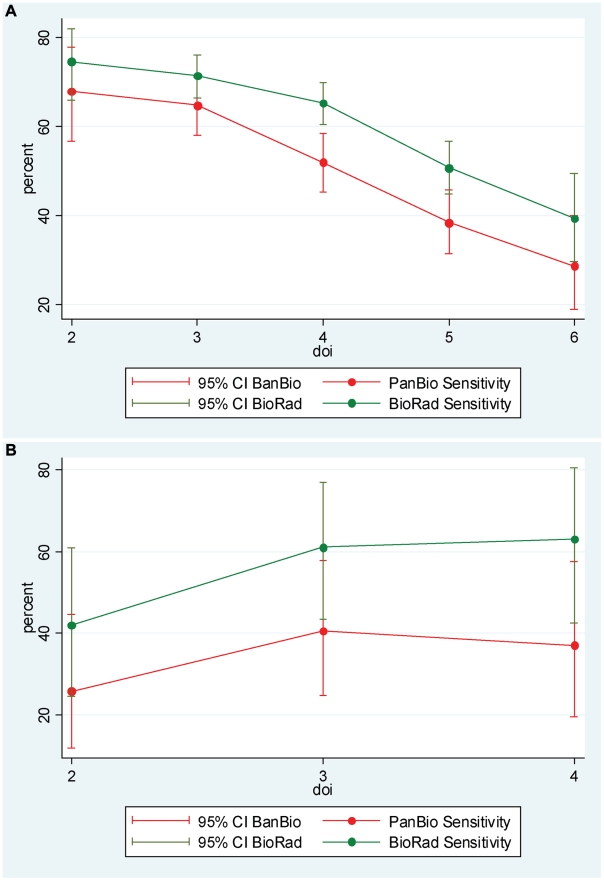
NS1 sensitivity by day of illness. (**A**) Shown is the sensitivity (±95% CI) of kits Pan-E and Platelia by day of illness in four Asian countries (N = 728 -kit Pan-E; N = 1152 -kit Platelia) amongst patients with a laboratory confirmed diagnosis of dengue where the acute sera were collected between day 2 and day 6 of illness. (**B**) Shown is the sensitivity (±95% CI) of kits Pan-E and Platelia in the first four days of illness in two Latin American countries (N = 93 -kit Pan-E; N = 90 -kit Platelia) amongst patients with a laboratory confirmed diagnosis of dengue where the acute sera were collected between day 2 and day 4 of illness. Data is presented for those days of illness with > = 40 observations respectively.

The sensitivity of both kits Pan-E and Platelia was influenced by the patient's duration of illness prior to test sample collection.. In Asian patients, kits Pan-E and Platelia were more sensitive in test samples collected early in the disease phase than at later time points (Figure 3A). The analysis was limited to days with more than 40 observations total which is why for Latin America only a narrow range of days can be shown (Figure 3B) and due to small sample size and large confidence intervals no trend is visible. A higher sensitivity of both NS1 detection assays were observed in Asian patients than in Latin-American patients at the first four days of illness (Figure 3B).

### NS1 sensitivity in relation to viral serotype

The sensitivity of each NS1 assay was considered in the context of the infecting serotype. Table 3 shows the sensitivity of kit Pan-E and Platelia assays according to DENV serotype as determined by RT-PCR or virus isolation. In our mainly hospital-based patient samples from 2006/2007 DENV-1 was most prevalent in Asia and DENV-2 most prevalent in Latin America (Table 4). For each of the four DENV serotypes kit Platelia had a greater sensitivity except for DENV-2, where the sensitivity was the same in both kits. In kit Platelia, sensitivity for DENV-2 was statistically significantly lower than for the other three serotypes pooled (DENV-2: 63%; 95% CI 57–69% versus 84%; 95% CI 82–88% for DENV-1, 3 and 4). The greater prevalence of DENV-2 in Latin American patients compared with Asian patients may help explain the lower sensitivity of both kit Pan-E and Platelia assays in Latin America (Figure 3B).

**Table 3:** NS1-sensitivity in the context of DENV serotype.

Serotype	Kit Pan-E		Kit Platelia	
	*N = 506	% Sensitivity (95%CI)	N = 862	% Sensitivity (95%CI)
DENV-1	223	79 (74–84)	415	87 (83–90)
DENV-2	169	62 (54–69)	257	63 (57–69)
DENV-3	87	60 (49–70)	142	82 (76–88)
DENV-4	27	52 (32–72)	48	79 (67–91)

*Number of DENV-positive samples by virus isolation or RT-PCR and serotype determined.

**Table 4:** Geographical and serotype stratification of the study population.

Country	DENV-1^a^	DENV-2	DENV-3	DENV-4	Total
Latin America					
**Nicaragua**	6% (2)	94% (32)	0	0	34
**Venezuela**	35% (19)	16% (9)	36% (20)	13% (7)	55
Mean	24% (21)	46% (41)	22% (20)	8% (7)	89
Asia					
**Malaysia**	62% (48)	9% (7)	17% (13)	13% (10)	78
**Thailand**	56% (86)	10% (15)	17% (26)	17% (26)	153
**Philippines**	0	10% (3)	87% (26)	3% (1)	30
**Vietnam**	47% (268)	35% (198)	10% (59)	1% (5)	568
Mean	48% (411)	27% (227)	15% (126)	5% (45)	829

a Percentages and absolute numbers (in brackets) of identified DENV serotypes by country.

### NS1 sensitivity in relation to IgM status

Detection of DENV-reactive IgM by MAC ELISA is the most commonly used approach to making a presumptive diagnosis of acute or recent dengue in endemic countries. Table 5 summarises NS1 sensitivity (kit Platelia assay only) in the context of IgM status and day of illness in confirmed dengue patients. The average sensitivity of NS1 testing in the first 7 days of sample collection was 65% (95%CI 62–69%) in acute samples where the IgM result was negative and 66% (95%CI 62–70%) when the acute test sample was IgM positive. Sensitivity figures increased to 74% and 70% if only samples collected in the first four days of illness were considered. Taking an algorithmic approach, when either the NS1 test or the IgM test on the acute sample was positive, the sensitivity for a presumptive (IgM) or definitive (NS1) diagnosis versus the reference result was 74% (95%CI 69–78) in samples collected at days 5 to 7. These figure increased to 82% (95%CI 79–84) in samples collected in the first four days of fever. These results suggested a combination of either IgM testing or NS1 testing (with kit Platelia) was sufficient to allow a presumptive (IgM) or definitive (NS1) diagnosis on an average of 82% of dengue cases enrolled in this study when acute early samples are tested. A similar analysis was performed with data obtained in the evaluation of kit Pan-E. Sensitivity figures of 66% (95%CI 60–72) in samples collected at days 5 to 7 and 71% in samples collected in the first four days of fever (95%CI 67–75) were obtained (Table S1).

**Table 5:** NS1 detection (kit Platelia assay only) in relation to IgM status and day of illness.*

Day of illness	Total no. of test samples	IgM positiveN =	% NS1 positive in IgM positive test samples [41]	IgM negativeN =	% NS1 positive in IgM-negative test samples [41]	% NS1 positive in total no. of test samples	% of test samples with a positive test (IgM or NS1) [41]**
Day 1	22	0	0 (0)	22	64 (14)	64(14)	64 (14)
Day 2	139	27	56 (15)	112	77 (86)	73 (101)	81 (113)
Day 3	372	123	78 (96)	249	71 (178)	74 (274)	81 (301)
Day 4	384	198	74 (146)	186	66 (122)	70 (268)	83 (320)
**Subtotal <5 days**	**917**	**348** ***	**74 (257)**	**569**	**70 (400)**	**72 (657)**	**82 (748)**
Day 5	256	125	54 (67)	131	52 (68)	53 (135)	75 (193)
Day 6	94	42	40 (17)	52	44 (23)	43 (40)	69 (65)
Day 7	6	4	75 (3)	2	0	50 (3)	67 (4)
Subtotal 5–7 days	356	171	51 (87)	185	49 (91)	50 (178)	74 (262)
**Total**	**1273**	**519** ***	**66 (344)**	**754**	**65 (491)**	**66 (835)**	**79 (1010)**

*Samples from 1273 patients with a confirmed dengue diagnosis between day of illness 1 to 7.

**Percentage of positives IgM samples plus positive NS1 samples of the IgM negative samples in the total tested samples.

***Percentages of IgM positive in total samples collected in the first four days (38%), days 5–7 (48%) and total (41%).

### NS1 sensitivity according disease severity

The sensitivity of each NS1 assay was considered in the context of disease severity and geographical region (Table 6). Cases were classified according the former WHO criteria for DF and DHF/DSS [27]. Sensitivity of kit Pan-E ranged from 29% (95%CI 12–46) in DF to 60% (95%CI 39–82) in DHF cases from Latin-American countries and from 50% (95%CI 43–57) in DF to 62% (95%CI 57–67) in DHF/DSS cases from Asia (overall sensitivity 47% in DF and 62% in DHF/DSS cases). The sensitivity of kit Platelia ranged from 41% (95%CI 28–55) in DF and 68% (95%CI 47–89) in DHF/DSS cases from Latin-American countries and 70% (95%CI 66–75) in DF and 68% (95%CI 64–72) in DHF/DSS cases from Asia (overall sensitivity of 68% for both DF and DHF/DSS total cases). Kit Pan-E showed higher figures of NS1 positive tests in severe cases, which are borderline statistically significant for Asia. Kit Platelia with overall higher sensitivity figures did not show a statistically significant association with disease severity.

**Table 6:** Sensitivity of Kit Platelia and Pan-E by geographic region and disease severity.*

	LAC**		SEA**		Total	
	DF***	DHF/DSS	DF	DHF/DSS	DF	DHF/DSS
Platelia	32/1341%(28–55)	22/1568%(47–89)	369/26070%(66–75)	628/42768%(64–72)	401/27368%(63–73)	650/44268%(64–72)
Pan-E	31/929%(12–46)	23/1460%(39–82)	228/11450%(43–57)	396/24562%(57–67)	259/12347%(41–54)	419/25962%(57–66)

*As indicated by the former WHO classification into DF and DHF/DSS for patients with NS1 test result and clinical classification available (N = 1051 for Platelia and 678 for Pan-E).

**LAC (Latin-American countries), SEA (Asian countries).

***N/positive NS1; % ; (95% CI).

### Overall specificity of NS1 tests versus reference diagnosis

The diagnostic specificity of kits Pan-E and Platelia assays was evaluated in 36 and 45 samples respectively from patients with no virological or serological laboratory evidence of acute or recent dengue. Both kits were negative in all these samples, which translates into a specificity of 100%.

### NS1 specificity in healthy blood donors and patients with other confirmed diagnoses

Since the number of patients with no evidence of acute or recent dengue was relatively small (n = 45) in this study, efforts were made to assess the specificity of dengue NS1 assays in patients with other confirmed infectious diseases whose transmission geographically overlaps with dengue, in healthy blood donors, and in blood donors with a serological history of DENV exposure. For the specificity analysis, a total of 304 sera were tested at two study sites (Cuba and Thailand). The specificity of kit Platelia was 100% in both sites whilst the kit Pan-E was 89% (Table 7). The lower specificity of kit Pan-E was in part due to false positive results in patients with Japanese encephalitis, Yellow Fever and acute Influenza.

**Table 7:** NS1 results as determined by Kit Pan-E and Platelia assays in control sera panels.

Panel 1 (Cuba)				Panel 2 (Thailand)			
		NS1 negative results				NS1 negative results	
Sera	N	Kit Pan-E	Kit Platelia	Sera	N	Kit Pan-E	Kit Platelia
Healthy blood donors	80	76	80	Acute Malaria sera	39	38	39
Cases with rash illness no dengue	10	8	10	Acute Leptospirosis sera	10	8	10
Acute Influenza sera	20	13	20	Acute Japanese Encephalitis sera	34	24	34
Acute RSV* sera	20	17	20	Acute Yellow Fever sera	15	12	15
Acute Hepatitis A sera	20	20	20	Dengue mono- or polyvalent immune sera (past infection)	27	27	27
**-**	**-**	**-**	**-**	Flavivirus non-immune sera	29	29	29
Total	150	134	150	Total	154	138	154
Specificity		90%	100%			90%	100%

*RSV (Respiratory Syncitial Virus).

## Discussion

Dengue is increasing in incidence globally and therefore accurate and efficient diagnostic tests are more important than ever for clinical care, surveillance support, pathogenesis studies and vaccine research. Diagnosis is also important for case confirmation, to differentiate dengue from other diseases such as leptospirosis, rubella, and other flavivirus infections, and for the clinical management and evaluation of patients with severe disease [16], [31]. The multicentre study described here assessed the diagnostic accuracy of two commercially available NS1 diagnostic tests. Two main findings were observed here: a) NS1 detection was overall only modestly sensitive for dengue diagnosis, with sensitivity highest in patients who presented early in their illness and b) a combined NS1 and IgM detection increased the overall sensitivity of dengue diagnostic.

The global dengue research agenda includes evaluating the validity, role and accessibility of available and new diagnostics of importance to reducing disease severity and case fatality [32]. Recognizing the importance of early diagnosis and taking advantage of the platform of the multicentre DENCO project, two commercial available NS1 detection ELISA kits (Pan-E Early Dengue, Panbio Ltd and Platelia™ Dengue NS1 Ag, Bio-Rad ), named here as kits Pan-E and Platelia, were evaluated in terms of sensitivity and specificity. Overall and within country sensitivity figures were higher for kit Platelia than kit Pan-E. With the exception of Nicaragua and The Philippines, sensitivity figures of kit Platelia varied from 64% to 76% while the sensitivity of kit Pan-E varied from 36% to 72%. Depending on the diagnostic method used for comparison, different figures of sensitivity of NS1 detection have been reported by others [12], [33], [34]. Kumarasamy et al., obtained an overall sensitivity of 93% using Platelia™Dengue NS1 Ag oscillating from 68% (in samples where the virus was isolated) to 90% in paired sera serologically confirmed as dengue [11], [35].

In the present study, relatively higher levels of sensitivity were observed in samples collected in the first four days of fever when samples from Asian patients were studied (interpretation limited for Latin America because of small sample size per day of illness).. Sensitivity was also higher in Asian patients compared with patients from Nicaragua and Venezuela. The small number of samples from Nicaraguan and Venezuelan patients (including a lower proportion of DHF/DSS cases) as well as the serotypes circulating could partially explain these observations (a high proportion of serotype 2 was found in Nicaraguan samples). The influence of duration of illness at the time of sample collection has been highlighted by others [6], [8], [10]. Figures of 93–100% sensitivity were obtained in samples collected at days 3 to 5 of fever [8] while others have reported figures higher than 85% in samples from day 1 to 3 in the Platelia assay [6], [11].

NS1 protein has been detected concomitant with viremia and coincident with the febrile stage [8].

In the present study, the highest sensitivity was obtained in RT-PCR positive samples. Sensitivity of kit Platelia in RT-PCR positive samples was 71% to 88% in Asian countries and 66% in Venezuela, but much lower in Nicaraguan samples (36%). Samples from this country were retested in a different laboratory by both NS1 detection kits but similar sensitivity results were observed (data not showed). The basis for low sensitivity in Nicaraguan samples remains unclear and will require further studies – but may partly be explained by the high proportion of serotype 2 in Nicaragua, which in both assays was associated with lower sensitivity. Indeed, as 94% (N = 32) of the serotypes recovered from Nicaragua were serotype 2, we cannot determine an estimate of sensitivity for the remaining 6% (N = 2).

Sensitivity varied by infecting serotype for each kit. The sensitivity of kit Pan-E was highest for DENV-1 infection (77%) and significantly lower for DENV-2 (60%), DENV-3 (57%) and DENV-4 (52%). The sensitivity of kit Platelia was also highest for DENV-1 infection (83%) and lowest for DENV-2 (60%). Consistent with DENV-1 infection being associated with high levels of NS1 detection, Xu et al., 2006, reported a sensitivity of 82% in an “in house” ELISA for the detection of NS1 protein of DENV-1 [36]. Similar results for the same serotype were reported by Alcon et al., 2002 [8]. The basis for different sensitivities for different serotypes requires further investigation. Potentially, this reflects different levels of avidity of the test mAbs for the relevant epitope(s) in NS1 from different serotypes, and potentially, different lineages from the same serotype. Also, this could potentially be related to the different sensitivities of the reference RT/PCR methods employed for dengue diagnosis. Alternatively, this might reflect different overall magnitudes of virus burden in patients with different serotypes. A relationship between NS1 detection and viraemia levels has been established previously [12], [15]. Since high early viraemia levels have also been linked to increased disease severity, it is plausible that NS1 tests are more sensitive in the first few days of illness in patients at risk of developing severe complications in their illness compared to patients with a more benign disease evolution. However, in our study, no association between NS1 detection and disease severity (indicated by classification of DF or DHF/DSS) was observed. Furthermore a regression analysis on NS1 positivity for DHF/DSS vs. DF (or severe vs. mild) and adjusted for serotype and for country was done and there was no effect seen (data not shown).

The specificity of NS1 tests could not accurately be estimated in the DENCO patient population as only a small number of cases had no serological or virological evidence of acute or recent dengue. Nonetheless, in patients who met our criteria for “not dengue”, the specificity of both NS1 test kits was very high (100%). To provide further insights into specificity, two sera panels from patients with other confirmed diagnosis and healthy individuals were tested. Kit Platelia showed the higher specificity (100%). Similar specificity values has been previously reported by others [10], [11], [15], [37]. The inclusion into the evaluating panel of samples from patients with acute Yellow fever and Japanese encephalitis virus infections suggest that no cross reaction among flaviviruses is observed with kit Platelia, however a larger number of samples collected from acute flavivirus infected patients need to be studied.

The dengue serotype, duration of illness prior to sample collection, and the presence of immunocomplexes (NS1-IgG) in previous dengue immune individuals could explain the low sensitivity observed in the Nicaraguan and The Philippines samples [38]. In the case of Nicaragua, DENV-2 was present in the 94% of the samples where the virus was identified by virus isolation and RT/PCR suggesting that this was the predominant serotype. The generally poor sensitivity for DENV-2 (60%) observed for both assays suggests this partially explains the low sensitivity in Nicaraguan samples [12]. In The Philippines, a conjunction of factors such as to the duration of illness prior to sampling and the high level of individuals with a secondary infection could partially explain the low sensitivity since high sensitivity was observed in RT-PCR positive samples (83%).

One of the limitations of our study is that it is heavily biased towards Asian patients and viruses, with 93% of the total samples coming from this region. The strengths of our study were that it was multicentre, prospective and encompassed a broad range of DENV serotypes and clinical presentations.

It is important to mention that no proficiency panel study on positive or negative samples was performed prior to evaluating the tested samples allowing us to have more comparable reference methods among participant laboratories. However protocols employed at each site, have been extensively evaluated previously [19]–[30]. In addition, the laboratories participants (including some WHO collaborating centres) are the reference centres for dengue diagnosis and laboratory surveillance in their respective countries and have participated in previous regional and international proficiency testing ([39], [40]

This study confirms and extends the findings of others in relation to the use of NS1 detections assays for the early diagnosis of dengue [6]–[12]. Although we could not study NS1 sensitivity and specificity in primary and secondary cases, in a small subset of samples classified as primary or secondary cases, a higher percentage of diagnose (90% over 80.6%) was obtained in the former (Vazquez S, manuscript in preparation).

In summary, we found the kit Platelia to be more sensitive and specific than kit Pan-E, with the sensitivity of both assays highest in the first few days of illness. Furthermore, we found that NS1 testing combined with IgM testing on the same test sample could yield a presumptive (IgM) or definitive (NS1) diagnose in as many as 82% of confirmed dengue cases using samples collected in the first four days of fever. As IgM detection is widely used for making a presumptive dengue diagnosis and in epidemiological surveillance, the use of a combined diagnostic algorithm including NS1 and IgM detection in samples collected in the first days of fever could provide clinically useful information to assist patient triage, management and outbreak response.

## Supporting Information

Checklist S1 STARD checklist.
(0.13 MB DOC)

Table S1 NS1 detection (kit Pan-E assay only) in relation to IgM status and day of illness.
(0.05 MB DOC)
