# Supplementary material for: Multi-Country Evaluation of the Sensitivity and Specificity of Two Commercially-Available NS1 ELISA Assays for Dengue Diagnosis
Source: PLoS Negl Trop Dis. 2010 Aug 31;4(8):e811. doi: 10.1371/journal.pntd.0000811 (PMC2930874; doi:10.1371/journal.pntd.0000811)
Supplement: Table S1 — NS1 detection (kit Pan-E assay only) in relation to IgM status and day of illness. (0.05 MB DOC) [file pntd.0000811.s002.doc]

**Table S1: NS1 detection (kit Pan-E assay only) in relation to IgM status and day of illness. ***

| Day of illness | Total no. of test samples | IgM positive  N= | % NS1 positive in IgM positive test samples [41] | IgM negative  N= | % NS1 positive in IgM-negative test samples [41] | % NS1 positive in total no. of test samples | % of test samples with a positive test (IgM **or**  NS1) [41]* * |
| --- | --- | --- | --- | --- | --- | --- | --- |
| Day 1 | 17 | 0 | 0 (0) | 17 | 53 (9) | 53(9) | 53 (9) |
| Day 2 | 104 | 23 | 43 (10) | 81 | 64 (52) | 60 (62) | 72 (75) |
| Day 3 | 243 | 72 | 78 (56) | 171 | 58 (100) | 64 (156) | 71 (172) |
| Day 4 | 226 | 98 | 59 (58) | 128 | 52 (66) | 55 (124) | 73 (164) |
| **Subtotal**  **<5 days** | **590** | **193***** | **64 (124)** | **397** | **57 (227)** | **59 (351)** | **71 (420)** |
| Day 5 | 174 | 71 | 31 (22) | 103 | 44 (45) | 39 (67) | 67 (116) |
| Day 6 | 77 | 35 | 23 (8) | 42 | 33 (14) | 29 (22) | 64 (49) |
| Day 7 | 6 | 4 | 50 (2) | 2 | 0 | 33 (2) | 67 (4) |
| Subtotal  5-7 days | 257 | 110 | 29 (32) | 147 | 40 (59) | 35 (91) | 66 (169) |
| **Total** | **847** | **303***** | **51 (156)** | **544** | **53 (286)** | **52 (442)** | **70 (589)** |

* In 847 patients with a confirmed dengue diagnosis between day of illness 1 to 7.

**Percentage of positives IgM samples plus positive NS1 samples of the IgM negative samples in the total tested samples.

***Percentages of IgM positive in total samples collected in the first four days (33%), days 5-7 (43%) and total (36%)
